# Combined approach of nanoemulgel and microneedle pre-treatment as a topical anticellulite therapy

**DOI:** 10.5599/admet.2461

**Published:** 2024-11-10

**Authors:** Hiba Imad Hameed, Mohammed Hussain Al-Mayahy

**Affiliations:** Department of Pharmaceutics, College of Pharmacy, Mustansiriyah University, Baghdad, Iraq

**Keywords:** Aminophylline, caffeine, cellulite, microneedles, nanoemulsions, tretinoin

## Abstract

**Background and purpose:**

Cellulite is caused by changes in the metabolism of the fatty tissue beneath the skin. Methylxanthines and retinoids are commonly added to the different anticellulite products. However, their topical permeation into the dermis is limited. Thus, the objective of this research is to formulate a nanoemulgel (NEG) containing a triple therapy of caffeine, aminophylline, and tretinoin as a topical anticellulite product to improve their skin permeation. Furthermore, the influence of microneedles (MNs) as skin pre-treatment on the permeation of the NEG was investigated.

**Experimental approach:**

Various nanoemulsion (NE) formulations were prepared using high-energy ultrasonication with different compositions and sonication amplitudes. Several characterisation tests were employed to select the optimum NE formulation. Then, the optimised NE formulation was incorporated with hyaluronic acid to prepare the NEG, which was, in turn, subjected to various evaluations. An *ex vivo* permeation study using human skin was performed for the NEG compared to a control preparation of plain gel. Additionally, a microneedling pen was applied as a skin pre-treatment at varying lengths prior to NEG application to examine its impact on the NEG’s permeation.

**Key results:**

The selected NEG has a homogenous and consistent texture with no coarse particles, a droplet size of 175.8 nm and polydispersity index (PDI) of 0.19, an optimum pH value of 5.28, high drug content of caffeine, aminophylline, and tretinoin (99.35, 98.48 and 98.05 %, respectively), high drug release values of approximately 100 % within 6 hours, appropriate viscosity, minimum skin irritation, and adequate short-term stability. The *ex vivo* permeation study showed that caffeine, aminophylline, and tretinoin permeated more and deposited in the skin with higher percentages from the NEG than plain gel. This skin deposition within the dermis was increased by applying the microneedling pen at varying lengths of 0.5, 1, and 2 mm as a skin pre-treatment.

**Conclusion:**

This combined approach of NEG formulation containing a triple therapy of caffeine, aminophylline, and tretinoin, along with MNs application, has the potential to serve as a topical anticellulite product, reducing cellulite formation and improving skin appearance.

## Introduction

The skin, the outermost covering of the body, plays a crucial role in maintaining psychological well-being and aesthetics [1,2]. As a result, there is a growing demand for cosmetic products, which are preparations used externally for cleansing, beautifying, promoting attractiveness, or altering appearance without affecting the body’s structure or functions. Cosmeceuticals, a hybrid product combining cosmetics with drugs, have emerged as a response to this demand [1,3]. Nanotechnology-based cosmeceuticals offer several benefits over traditional cosmeceuticals, including the small particle size and high surface area. Thus, nano-cosmeceuticals can improve contact time with the application site and enhance the penetration of active molecules into the skin [3]. The most common types of nanocarriers being investigated for topical drug and cosmeceutical delivery include solid lipid nanoparticles, polymeric nanoparticles, nano-vesicular carriers, microemulsions, and nanoemulsions [4]. Nanoemulsions (NEs) are currently the subject of significant research due to their potential to transport various drugs and improve therapeutic outcomes [5]. NEs are nanocarrier systems composed of two immiscible liquids, oil and water; one of them is dispersed as nanodroplets in the other [6], with mean droplet size ranging from several tens to several hundred nanometres stabilised by an emulsifier [7]. An emulsifier, a type of surfactant, is adsorbed at the interface between the dispersed and continuous phases, reducing the surface tension and effectively stabilizing the system [8]. The formation of NEs involves a two-stage procedure. Initially, coarse emulsions are created and subjected to ultrasonication or high-pressure homogenization. This step breaks down the large droplets into nanosized droplets, resulting in the formation of NEs. Since NEs are composed of two phases, water and oil, they can be used to deliver both hydrophilic and hydrophobic drugs in the same dosage form [7]. However, the NEs’ low viscosity leads to inadequate retention of the formulation on the skin. To address this, a gelling agent can be added to the NE, resulting in the formation of a nanoemulgel (NEG). Aside from improving drug penetration through the skin, it is also critical to maintain therapeutic concentrations at the application site for an extended length of time. The gel portion increases formulation viscosity, resulting in a longer retention time at the area of application [8].

This study focuses on cellulite, a topographic change caused by female sex hormones, characterised by an orange peel appearance of the skin. Cellulite affects 85 to 95 % of women and is observed in the pelvic region, abdomen, and lower extremities [9-11]. The hypodermis, the deepest layer of the skin, stores most body fat. However, changes in adipose tissue, disruption of microcirculation, and weakening of connective tissue can form large fat globules that expand towards the dermis, resulting in dimpled skin [12]. Numerous treatment options have been suggested to address cellulite. Topical treatment is convenient and advised for the management of mild to moderate conditions and as a complementary treatment for severe cellulite. Additionally, oral supplements, radiofrequency, and surgical procedures are also available [13]. The most preferred cosmeceutical active ingredients for treating cellulite are methylxanthine derivatives, such as caffeine and aminophylline [14]. They can improve adipocyte lipolysis by inhibiting phosphodiesterase, raising cyclic adenosine monophosphate (AMP), and enhancing dermal microcirculation [15,16]. Retinoids, such as tretinoin, effectively increase the dermal content and architecture of collagen and dermo-epidermal proteins, which aid in rebuilding the normal structure of the dermis and subcutaneous tissue [16]. However, effective treatment requires the active molecules to cross the *stratum corneum* barrier and reach the dermis and hypodermis in sufficient concentration [17]. One of the active methods to improve the delivery of therapeutics into the skin is via the use of microneedles (MNs).

MNs are tiny micron-sized needles (25 to 2000 μm in height) that overcome the *stratum corneum* barrier by piercing the skin to create micro-conduits through which drugs can be more easily transported into/across the skin. They are generally considered a minimally invasive, painless technique with self-administration ability in comparison with hypodermic injections [18,19]. MNs can be categorised into five types: solid, hollow, coated, dissolving, and hydrogel MNs. The skin pre-treatment approach is frequently achieved using solid MNs (poke and patch), which puncture the skin, followed by drug application. This provides a potential opportunity for improving drug permeability into the skin via the created microchannels [20]. Previous studies have demonstrated that skin pre-treatment by solid MNs, such as those that used a microneedling pen with oscillating properties, could enhance drug delivery into the skin [21,22]. We propose using a microneedling pen as a physical enhancement technique, which would be a suitable strategy for cellulite treatment. The pen can be conveniently applied over the large cellulite area, and simultaneously, the microneedling technique could initiate skin rejuvenation and improve skin appearance by stimulating the skin’s natural healing mechanism [23,24]. Thus, the aim of this research is to improve the skin permeation of caffeine, aminophylline, and tretinoin via a nanoemulgel formulation as a topical anticellulite product, as well as to investigate the influence of MNs as a skin pre-treatment on the permeation of the nanoemulgel. To the best of our knowledge, this work demonstrates for the first time a new approach in the topical treatment of cellulite that combines the formulation of a triple therapy nanoemulgel along with the use of MNs.

## Experimental

### Materials

Caffeine (anhydrous) was kindly received as a gift sample from pioneer company for pharmaceutical industries, Iraq. Aminophylline was purchased from Macklin Biochemical Co., Ltd., China. Tretinoin and sodium oleate were purchased from Bide Pharmaceutical Technology Co., Ltd., China. Polyoxyethylene sorbitan monolaurate (Tween 20), and polyoxyethylene sorbitan monooleate (Tween 80) were purchased from HiMedia Laboratories Pvt. Ltd., India. Cetyl alcohol (98 %, Alfa-Aesar, Kandel, Germany). Oleic acid was supplied from Thomas Baker (chemicals) Pvt Ltd, India. Hyaluronic acid (molecular weight 250 KD) was purchased from Shandong Bouliga Biotechnology Co., Ltd. China. Hydrochloric acid was supplied from Thomas Baker (Chemicals) Pvt Ltd, India. All other oils and chemicals were of analytical reagent grade and deionised water was used for all experiments. A microneedling pen was purchased from ENZO Professional Electronic Co., Limited, Italy. This device has twelve tapered conical needles, a base diameter of 370 μm, a tip diameter of 53.4 μm, and five different oscillation speeds. The needle length can be adjusted between 250 to 2000 μm.

### Solubility study

The saturated solubility study of caffeine, aminophylline, and tretinoin in different solvents was performed to select the most proper solvent for each drug to be used in the development of NEs. The solubility was performed by adding an excess amount of each drug to 3 mL of each solvent (deionised water, oleic acid, castor oil, grape seed oil, mint oil, jojoba oil, vitamin E oil, Tween 80, ethanol, acetate buffer pH 5.5 with 2 % Tween 80, phosphate buffer pH 7.4, and phosphate buffer pH 7.4 with 2 % Tween 80) in a stoppered glass vial. The glass vials containing the mixtures were then placed on a magnetic stirrer with a rotation rate of 600 rpm at room temperature for 72 hours to ensure equilibrium. After that, the stoppered vials were centrifuged at 3500 rpm for an hour, and the supernatant was then filtered through a 0.45 μm syringe filter. Following this, the filtrate was appropriately diluted with the mobile phase to be analysed by HPLC to determine the AUC of the eluted samples’ peaks [25].

### Preparation of nanoemulsion formulations

A high-energy ultrasonication method was used to prepare an oil in water (o/w) NEs. First, the aqueous phase and oil phase were prepared separately. In brief, the oil phase was prepared by solubilizing 0.05 wt.% tretinoin in oleic acid using a magnetic stirrer at 600 rpm, then cetyl alcohol 1 wt.% was added to it and mixed until dissolved. The aqueous phase was prepared by dissolving a surfactant mixture (Tween 20 and sodium oleate) at different concentrations (5, 7.5, 10, 12.5 and 15 wt.%) in deionised water. The surfactant mixture was chosen according to the required hydrophilic-lipophilic balance (HLB) value of the oil phase. After that, 3 wt.% of caffeine and 2 wt.% of aminophylline were added and mixed at 600 rpm until dissolved. The pH of the aqueous phase was then adjusted to about 5 by adding a few drops of 1 M HCl. The oil phase was gradually added to the aqueous phase and mixed for 30 minutes. Then, the formulations were subjected to ultrasonication using a probe sonicator at 50 Hz for 20 minutes at different amplitudes (20, 40 and 60 %) to obtain the NEs. The composition of the NE formulations is listed in Table 1. During the ultrasonication process, there was heat generation, and this problem was addressed by placing the formulations in an ice bath [26].

**Table 1.** The composition of the nanoemulsion formulations with process variables

Formulation code	Content, wt.%				Sonication amplitude, %	Time, min
	Oleic acid	Tween20/sodium oleate	Cetyl alcohol	Water		
NE1	5	5.0	1	89.0	20	20
NE2	5	5.0	1	89.0	40	
NE3	5	5.0	1	89.0	60	
NE4	5	7.5	1	86.5	20	20
NE5	5	7.5	1	86.5	40	
NE6	5	7.5	1	86.5	60	
NE7	5	10.0	1	84.0	20	20
NE8	5	10.0	1	84.0	40	
NE9	5	10.0	1	84.0	60	
NE10	5	12.5	1	81.5	20	20
NE11	5	12.5	1	81.5	40	
NE12	5	12.5	1	81.5	60	
NE13	5	15.0	1	79.0	20	20
NE14	5	15.0	1	79.0	40	
NE15	5	15.0	1	79.0	60	

### Characterisation of nanoemulsions

The mean droplet size and polydispersity index (PDI) for the NE formulations were determined using the dynamic light scattering technique (ZS90; Malvern Instruments Ltd., Malvern, UK). The formulation was tested for light scattering at 25 °C and a 90° angle without dilution [27]. The effect of the surfactant mixture concentration and the sonication power amplitude on the droplet size of the NEs was investigated [28]. The zeta potential of the optimum NE was measured using the electrophoretic light scattering method. The sample was diluted at a ratio of 1:100 by deionised water and then placed in zeta cells for measurement (ZS90; Malvern Instruments Ltd., Malvern, UK) [29].

A calibrated pH meter was used to measure the pH of the NEs by immersing the instrument bulb into each formulation. The drug content of NEs was determined by weighing approximately 1 g of each formula containing (30 mg of caffeine, 20 mg of aminophylline, and 0.5 mg of tretinoin) and diluted it with 100 mL deionised water to be measured by the HPLC [30]. A Brookfield digital viscometer (LVDV-E, USA) with spindle No. 61 was used to determine the NEs viscosity at room temperature. The spindle was inserted into 30 mL of the formulation for 3 minutes at a rotational speed of 60 rpm [31]. These tests were performed in triplicate, and the results were obtained as a mean ± SD.

The *in vitro* drug release for NEs was performed using Franz diffusion cells (*n* = 3) with a 0.785 cm^2^ surface area. As a receptor fluid, 10 mL of phosphate buffer pH 7.4 with 2 % Tween 80 was added to the receptor chamber. A cellulose nitrate membrane with a pore size of 0.45 μm was used as a release membrane, which was sandwiched between the donor and receptor chambers by a clamp. Franz cells were later immersed in the diffusion cell apparatus at 37 °C, and 1 g of the selected NE formulations was applied in the donor chamber. A small magnetic bar stirred the receptor fluid continuously at 100 rpm. The sink condition was preserved by compensating the 0.5 mL sample withdrawn from the receptor fluid with the same volume of preheated, fresh receptor fluid. HPLC analysis of the withdrawn samples was performed to determine the percentage of drug released [32-34].

The selection of the optimum NE formulation was based on the droplet size, PDI, viscosity, drug content, pH, and drug release study results. The optimum NE formulation was then used to prepare NEGs. The morphology of the optimum NE was examined using the field emission scanning electron microscopy technique (FE-SEM). The FE-SEM image was captured using Xt microscope control software.

### Preparation of nanoemulgels

The total amount of deionised water required for the preparation of a NEG was divided into two parts: one part was used to prepare the NE formulation, and the other is used for the preparation of the gel base [8]. Hyaluronic acid was used to prepare the gel base at three different concentrations of 1, 1.5 and 2 wt.% by dispersing it in deionised water with stirring at 600 rpm, 37 °C until completely dissolved. After that, the pre-prepared NE was added drop by drop to the gel base and homogenized until an NEG was formed.

For the preparation of the plain gel, the active ingredients (3 wt.% of caffeine, 2 wt.% of aminophylline, and 0.05 wt.% of tretinoin) were dissolved in phosphate buffer pH 7.4 with 2 % Tween 80. The pH of this solution was then adjusted to the pH 5 using 1N HCl. After that, 1.5 wt.% of hyaluronic acid was used to prepare the gel base. Finally, the drug solution was added gradually to the gel base while stirring on a magnetic stirrer until a homogeneous gel was obtained [35].

### Characterisation of nanoemulgels

Visual examination of the general appearance of the prepared NEGs was performed. This includes the examination of the colour, homogeneity, and consistency [31]. A small amount of the formulations was placed between the thumb and index finger to check their texture, consistency, and homogeneity [36]. The spreadability value of the NEG formulation was determined by measuring the spreading diameter of 1 g of the gel between two horizontal glass plates (20×20 cm) before and after the application of 500 g load for 5 minutes [37]. This test was performed in triplicate and the results were obtained as a mean ± SD. Other tests for determining droplet size, zeta potential, pH, drug content, viscosity, and drug release for the selected NEG formulation were performed using a similar procedure mentioned previously in the characterisation of NEs.

### Ex vivo permeation and skin deposition study

Franz diffusion cells were utilised to determine the permeation of caffeine, aminophylline, and tretinoin from the NEG and plain gel formulations through the excised human skin. Full-thickness human skin was collected from a 35-year-old woman who had undergone abdominal plastic surgery. The patient signed a consent form to collect and use her skin in laboratory experiments. To prepare the skin for the permeation study, the skin was first cleaned with water and normal saline and then dried using tissue paper. The subcutaneous fatty layer was removed by a scalpel, and the skin samples were then enveloped in an aluminium foil and stored at -20 °C until needed. The skin was utilised within one month of being frozen. Briefly, the permeation study was carried out using Franz diffusion cells with a surface area of 0.785 cm^2^. The human skin was mounted between the donor and receptor chambers with a clamp. The receptor chamber was filled with 10 mL of phosphate buffer pH 7.4 with 2 % Tween 80 as a receptor fluid. Franz cells were then immersed in a diffusion cell apparatus at 37 °C with a stirring speed of 100 rpm for 24 hours. One gram of the NEG was placed on the surface of the skin in the donor chamber, and an aliquot of 0.5 mL was withdrawn from the receptor chamber at different time intervals (1, 2, 3, 4, 5, 6, 8, 12 and 24 hours). The withdrawn samples were replaced with a fresh preheated medium to preserve the sink condition. The withdrawn samples were then analysed by HPLC to measure the drug concentration [38]. The plain gel was tested for skin permeation under the same experimental conditions as the NEG formula for comparison.

Besides the determination of the drug’s amount present in the receptor fluid, the amount of drug deposited within the skin was also measured. The skin was removed from the Franz cells following the completion of the *ex vivo* permeation test, and it was cut into small pieces that were placed in glass vials. After that, 10 mL of phosphate buffer pH 7.4 with 2 % Tween 80 was added to these vials and sonicated in a bath sonicator for 2 hours and then left overnight. Liquid samples withdrawn were filtered through a 0.45 m Millipore filter, suitably diluted, and analysed by HPLC to estimate the percentage of drug deposition within the skin [32,39,40]. Additionally, the excess formulation of the NEG and plain gel present on the skin surface and on the wall of the Franz cells was removed by wiping it with a piece of cotton (cotton swab). After that, the cotton was placed in a stoppered glass vial, soaked in 10 mL of phosphate buffer pH 7.4 with 2 % Tween 80 and sonicated in a bath sonicator for 2 hours, and left overnight. Following this, the samples were filtered by 0.45 m Millipore syringe filter, suitably diluted and analysed by HPLC to determine the amount of drug retained over the skin surface, *i.e*., skin wash (not permeated).

### Skin pre-treatment with microneedles

To further examine the impact of MNs on the NEG permeation profile, an additional permeation study was conducted. The permeation study was carried out as previously discussed in the “*Ex vivo* permeation and skin deposition study” section, except the skin was prepared for this experiment by placing it on a flat desk and applying the microneedling pen vertically to the skin. Twelve solid (metal) microscale needles are contained within the microneedling device. The microneedling pen was applied at varying lengths of 0.5, 1 and 2 mm, with a minimum vibration speed of 4000 rpm. A gentle application of pressure (thumb pressure) was utilised to maintain the application time at one minute. Following this, the skin samples were affixed to Franz cells in an upward-facing orientation with the *stratum corneum* and dosed with the NEG formulation [21].

Furthermore, to investigate the amount of drug deposited within each skin layer (epidermis and dermis), the heat-separation technique was used to separate the epidermis layer from the dermis. In this case, following the skin removal from Franz cells, it was soaked for 1 minute in preheated deionised water at 60 °C. The epidermis was then separated from the dermis using tweezers and kept in glass vials. The remaining skin (dermis) was cut into small pieces and placed in glass vials. Then the epidermis and dermis were soaked in 10 mL phosphate buffer pH 7.4 with 2 % Tween 80, sonicated in a bath sonicator for 2 hours and left overnight. Liquid samples were then withdrawn, filtered through a 0.45 pm Millipore filter, suitably diluted and analysed by HPLC to estimate the percentage of drug deposition within each layer [32,39].

### Skin irritation test and stability study of the nanoemulgel

The protocol of the skin irritation test was reviewed and approved by the animal ethical committee of Mustansiriyah University/College of Pharmacy (number 27/2024) for utilising Wistar Albino rats for the skin irritation test. The skin irritation test was conducted in male Wistar rats (*n* = 5) aged 2 to 3 months, weighing 200 to 250 g. Their skin appeared normal, and they had no visible wounds or scratches. The hair on the dorsal side of the rat was shaved using clippers one day before starting the experiment [41]. Wister rats were divided into three groups. Group 1 was the positive control group (formalin group, used 0.8 vol.% aqueous solution of formalin), group 2 was the negative control group (untreated group), and group 3 was the test group (NEG group). NEG formulation was applied on the hair-free skin of the rat by spreading it evenly over the tested area. The skin erythema and oedema were assessed at 1, 24, 48 and 72 hours following the topical application of the formulation.

Regarding the stability study of the NEG, the formulation was stored for three months in a sealed glass vial away from light at 4±2, 25±2 and 40±2 °C. At regular times (0, 1, 2 and 3 months), the NEG was examined visually, and samples were withdrawn and analysed for pH, particle size, polydispersity index, and drug content [42].

### HPLC analysis

The chromatography of caffeine, aminophylline, and tretinoin was performed by a modified gradient HPLC method using the SYKAM HPLC system (Germany) with a C18-ODS (25×4.6 mm) column as the stationary phase. The column oven temperature was maintained at 30 °C, and the injection volume was 100 μL. The mobile phase A was deionised water with 1 % acetic acid, and the mobile phase B was methanol. The flow rate was 0.8 mL/minute. The gradient HPLC method timescale is illustrated in Table 2.

**Table 2.** The gradient HPLC method timescale for the separation of caffeine, aminophylline, and tretinoin

Time, min	Content, vol.%		*λ*_max_ / nm (UV detector)
	Mobile phase A (water + 1 % acetic acid)	Mobile phase B (methanol)	
0-4	90	10	356
4-7	25	75	264
7-15	30	70	264

### Statistical analysis

Statistical analysis was carried out using t-test, one-way, and two-way ANOVA, followed by the least significant difference (LSD), which was used to compare between groups in this study. The results are presented as a mean ± SD with *P* values of ≤ 0.05 regarded as statistically significant.

## Results and discussion

### Solubility study

The results obtained from the saturated solubility study of caffeine, aminophylline, and tretinoin are illustrated in Table 3. Caffeine and aminophylline showed higher solubility in deionised water at 39.5 and 34.9 mg/mL, respectively, than other solvents. This is due to their hydrophilic nature. Therefore, deionised water was used as a solvent for caffeine and aminophylline to dissolve the required dose in the aqueous phase of the prepared NEs. Since tretinoin is a water insoluble substance, its solubility was investigated in different oils. Among all the oils that were evaluated, the highest solubility of tretinoin was observed with oleic acid of 39.6 mg/mL; thus, it was selected as the oil phase of the prepared NEs. The HPLC chromatogram of the mixture of three drugs (caffeine, aminophylline, and tretinoin) is shown in the Supplementary material, Figure S1.

**Table 3.** The saturated solubility values of caffeine, aminophylline, and tretinoin in various solvents

Sample solution	Solvent used	Concentration, mg/mL		
		Caffeine	Aminophylline	Tretinoin
1	Deionised water	39.5	34.9	
2	Ethanol	20.5	15.0	8.6
3	Phosphate buffer pH 7.4	30.4	29.4	9.2
4	Phosphate buffer pH 7.4 with 2 % Tween 80	37.0	74.0	14.0
5	Oleic acid			39.6
6	Mint oil			7.5
7	Grape seeds oil			7.0
8	Castor oil			9.0
9	Jojoba oil			1.9
10	Vitamin E oil			8.6

Additionally, it is necessary to find a universal solvent that can solubilize the three drugs together to be used as a receptor fluid. Accordingly, caffeine, aminophylline, and tretinoin showed adequate solubility in phosphate buffer pH 7.4 with 2 % Tween 80 (37, 74 and 14 mg/mL, respectively). Therefore, it was selected as a receptor fluid for the *in vitro* drug release and *ex vivo* permeation studies to maintain the sink conditions and ensure continuous drug release and permeation through the skin during the experimental work.

### Preparation of nanoemulsion formulations

The surfactant mixture was chosen based on the required HLB (HLBr) value of the oil phase, combining binary surfactants with higher and lower HLB values than the HLBr value of oleic acid. Binary surfactants, Tween 20 with an HLB value of 16.72 [43], and sodium oleate with an HLB value of 18 [44] were used to achieve the HLBr value of oleic acid of 17 [45]. The fraction of each surfactant is calculated using Equation 1.


HLBr_oleic acid_ = *X* HLB_Tween 20_ + (1-*X*) HLB_sodium oleate_ (1)

When the HLB values of the oil phase and the surfactant system are well-matched, stable emulsions are usually formed [46]. A stable emulsion could be formulated with a blend of surfactants compatible with the required HLB value of the oil phase used [47]. The use of surfactant mixtures increases the repulsive interactions of particles in the emulsion and, consequently, improves their flocculation stability [48]. However, our observation revealed that the stability of the prepared NEs was only maintained for a few days following preparation, and then they underwent a phase separation phenomenon. This indicated the need for an additional stabilizer (co-stabilizer) to further enhance the stability of the NEs for a longer period. In this context, cetyl alcohol was added at a concentration of 1 wt.% to investigate its role in improving stability. Cetyl alcohol is a high molecular-weight alcohol used as a thickening agent in topical preparations to prevent phase separation and increase product stability [49]. The images of NEs prepared with and without cetyl alcohol are illustrated in the supplementary material, Figure S2. Another challenge that faced the preparation of stable NEs was the change of tretinoin colour from light yellow to amber during storage, which suggests the likelihood of tretinoin oxidation (photo instability). It was found that this oxidation reaction is pH-dependent and catalysed by the alkaline medium of the prepared NEs, which have a pH value of 8, due to the basic nature of caffeine and aminophylline that are dissolved in the aqueous phase of the NEs. Thus, to overcome this obstacle, the pH of the NEs was made slightly acidic at different pH values (4, 5, and 6) by the addition of a few drops of 1 N HCl to select the optimum pH value for the NEs stability. A NE prepared at pH 6 exhibited a minor colour change in comparison to the formulations prepared at pH 4 and 5, which demonstrated greater colour stability. Therefore, pH 5 was selected for the preparation of NE formulations as it closely resembles the skin’s pH. The images of the NE formulations prepared at different pH values are demonstrated in the supplementary material, Figure S3.

Fifteen NE formulations were prepared by the high energy ultrasonication method using different percentages of the surfactant mixture. Additionally, the impact of processing factors, such as the sonication amplitude, on reducing the droplet size of the prepared NEs was investigated. All the prepared formulas were characterised and evaluated to select the optimum NE formulation.

### Characterisation of nanoemulsions

The NEs’ droplet size and PDI are displayed in Table 4. The droplet size is affected by the formulation variables, such as the concentration of surfactant mixture used, and by the processing parameters, such as the value of the energy input [50]. The prepared NEs had a droplet size ranging from 133.6 to 566.3 nm. The NE formulas with a smaller droplet size are preferred due to their faster release rate and a greater opportunity to penetrate the deeper layers of the skin; for instance, 600 nm nanodroplets can only remain on the *stratum corneum* surface, while droplets with a size of 300 nm can reach the skin’s deeper layers [51]. Several studies also reported that NEs with a droplet size below 500 nm are suitable for topical delivery [52]. Thus, most formulations were considered to fall into the acceptable nanosized range. The polydispersity index (PDI) determines the measure of droplet size homogeneity. The PDI values range from 0.0 to 1.0 [53]. The smaller PDI values indicate a homogenous distribution of droplet size, while higher values suggest a broader distribution. The PDI values obtained for all of the NEs were in the range of 0.21 to 0.31, showing a narrow and uniform size distribution [52].

**Table 4.** The droplet size and PDI values of the prepared nanoemulsions

Formulation code	Droplet size, nm	PDI	Formulation code	Droplet size, nm	PDI
NE1	154.8	0.21	NE9	169.1	0.24
NE2	140.7	0.22	NE10	557.9	0.26
NE3	133.6	0.22	NE11	306.9	0.31
NE4	194.4	0.24	NE12	192.8	0.25
NE5	163.6	0.26	NE13	566.3	0.22
NE6	160.2	0.24	NE14	341.7	0.30
NE7	289.0	0.25	NE15	225.5	0.24
NE8	229.0	0.26			

As shown in Table 4, increasing the surfactant concentration from 5 to 15 % resulted in an increase in the NE’s droplet size. NE1, NE2, and NE3, with a surfactant concentration of 5 wt.% had a droplet size of 154.8, 140.7 and 133.6 nm, respectively, while NE13, NE14, and NE15 possessed 15 wt.% surfactant concentration had 566.3, 341.7 and 225.5 nm, respectively. This is most likely due to the presence of extra-free surfactant molecules that were high enough to cover the oil droplets completely. The droplet size of the NEs would consequently increase as a result of excess surfactant molecules forming aggregates in the continuous phase [54]. Thus, by decreasing the surfactant concentration, the incorporation of water into oil droplets was improved, causing the splitting of the droplets and resulting in a smaller droplet size [55,56].

The droplet size of the prepared NEs was also shown to decrease with the increase in the amplitude of the probe sonicator used to prepare these formulations. As seen in Table 4, formulations prepared with 60 % amplitude had a smaller droplet size than formulations prepared with 40 %, and in turn, formulations prepared with 40 % amplitude were smaller than formulations prepared with 20 %. This might be due to more energy input from the probe sonicator, which could generate stronger cavitation and shear stress, causing the emulsion to break into smaller droplets during the preparation of the NEs [57]. Therefore, NE formulations prepared with 60 % amplitude (NE3, NE6, NE9, NE12, and NE15) were selected for further evaluation tests and investigations, including the measurement of the pH, drug content, viscosity, and drug release study.

The pH values of the selected NEs (NE3, NE6, NE9, NE12, and NE15) were within the range of 4.92 to 5.31, as shown in Table 5. The measured pH values of NEs were close to the skin’s pH of 4.5 to 6.5. This implied they could be applied to the skin without causing irritation [58]. The drug content determination of the selected NEs, as illustrated in Table 5, revealed a high drug content of 97.75 to 99.94 % for the three drugs, which is within the USP accepted range of 85 to 115 %. This indicates the suitability and reliability of the preparation method. The viscosity measurement for the selected NE formulations is shown in Table 5. The prepared formulas have viscosity results ranging between 50.6 ± 0.529 and 75.90 ± 0.360 mPa·s (cp). It was observed that as the surfactant concentration increased, the viscosity also increased. This could be due to the entrapment of water molecules within the cross-linked surfactant chains, as well as the fact that a higher concentration of the surfactant would make the dispersion medium less flexible [59].

**Table 5.** The pH, viscosity and drug content values of the selected NE formulations; the results are represented as a mean ± SD, *n*=3

Formula code	pH	Viscosity, mPa·s (cp)	Drug content, %		
			Caffeine	Aminophylline	Tretinoin
NE3	5.31 ± 0.035	50.60 ± 0.529	99.94 ± 0.031	99.50 ± 0.051	98.88 ± 0.046
NE6	5.12 ± 0.031	56.80 ± 0.400	99.23 ± 0.241	98.37 ± 0.068	98.77 ± 0.127
NE9	4.92 ± 0.021	63.00 ± 0.781	99.42 ± 0.595	99.63 ± 0.029	97.70 ± 0.115
NE12	5.23 ± 0.025	67.16 ± 0.378	99.23 ± 0.654	99.16 ±0.085	97.75 ± 0.035
NE15	5.17 ± 0.047	75.90 ± 0.360	99.30 ± 0.030	99.48 ± 0.190	98.63 ± 0.116

**Figure 1.**
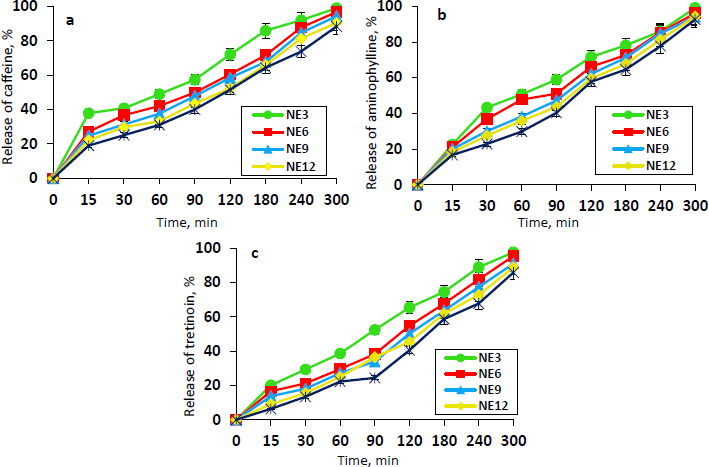
The release profiles of (a) caffeine, (b) aminophylline, and (c) tretinoin from the NE formulations (NE3, NE6, NE9, NE12, and NE15) in phosphate buffer pH 7.4 with 2 % Tween 80

**Figure 2.**
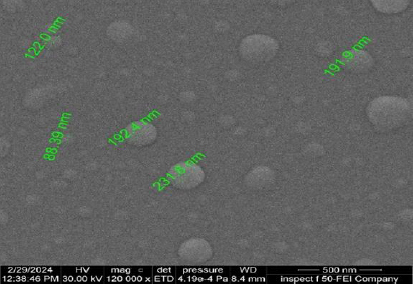
The FE-SEM image of the optimum nanoemulsion formulation NE3

The *in vitro* release profiles of caffeine, aminophylline, and tretinoin from the selected NEs (NE3, NE6, NE9, NE12, and NE15) are shown in Figures 1a, b, and c, respectively. The release values from the different NE formulations following 5 hours were high in the range of 88.24 to 98.87 % for caffeine, 92.57 to 99.12 % for aminophylline, and 85.77 to 97.51 % for tretinoin. The NE3 formulation demonstrated the highest release percentage of caffeine, aminophylline, and tretinoin (98.87, 99.12 and 97.51 %, respectively), which is statistically significant (*P* ≤ 0.05) from other formulations. Presumably, this could be related to its smaller mean droplet size and lower viscosity compared to other NE formulations. The NE3 was selected as the optimum formulation since it showed the minimum droplet size, acceptable PDI, adequate viscosity, higher drug content, appropriate pH, and maximum drug release. Therefore, it was chosen for the preparation of the final dosage form ‘nanoemulgel’. The morphology of the NE3 droplets was examined using FE-SEM imaging. As evident from Figure 2, the FE-SEM image confirmed the presence of the NE3 formulation in nanosized droplets. The average droplet size is approximately 165.29 nm with a spherical, non-adherent shape.

### Preparation of nanoemulgels

The NEGs were prepared by the incorporation of the optimum NE formulation (NE3) into an aqueous dispersion of hyaluronic acid (HA) at different concentrations of 1, 1.5 and 2 wt.%. HA was selected as a gelling agent for the preparation of the NEG since it produced a homogenous gel with no coarse particles and proper consistency [60]. In addition, HA is considered a natural endogenous component of the skin that can aid in improving skin appearance [61]. Moreover, for comparison with the NEG, a plain gel containing the same drugs’ concentration was prepared to demonstrate the impact of the nanodroplet size of the NEG and the influence of its components of oleic acid and surfactant mixture, which act as penetration enhancers, on permeation and deposition in the skin layers.

### Characterisation of nanoemulgels

The examination of the general appearance of the prepared NEGs showed that the NEG with 1 % HA had a low viscosity and was almost fluidic, while the NEG with 2 % HA had a high viscosity and coarse particles that were difficult to apply. The most appropriate concentration of HA that was used to prepare the NEG was 1.5 %, which resulted in a homogeneous, consistent, easy-applied creamy yellow NEG free of coarse particles. Therefore, it was selected for further evaluation tests and investigations. The images of the NEGs prepared at different concentrations of HA are shown in the supplementary material, Figure S4. The spreadability factor is one of the qualities to consider when developing a semisolid pharmaceutical formulation for skin application [62]. Appropriate spreadability helps achieve a uniform application of the topical gel, representing the size of the area over which the gel spreads [63]. The spreadability of the NEG was increased by 1.66 ± 0.236 cm after the application of 500 g weight. The images of the spreadability test of the NEG are illustrated in the Supplementary material, Figure S5.

The droplet size and PDI measurement results of the NEG and plain gel are illustrated in Table 6. The results demonstrated a significant increase in the droplet size value of the plain gel of 1241 nm in comparison with 175.8 nm of the NEG. The smaller size of the NEG droplets confirms the impact of the high-energy ultrasonication method employed in the production of nanosized droplets of the NEG since the plain gel was prepared without using this method. The droplet size distribution of the NEG and plain gel is illustrated in the Supplementary material, Figure S6. In comparison with the NE, the droplet size of the NEG was larger than that of the corresponding nanoemulsion formulation, NE3, which had a droplet size of 133.6 nm. This increase in the droplet size may be attributed to the presence of the gelled hyaluronic acid, which entrapped the nanodroplets within the polymeric matrix [64]. The NEG’s PDI value of 0.19 was within the acceptable range, and the addition of hyaluronic acid to prepare the NEG did not result in a significant difference from the PDI of the NE3 of 0.22.

**Table 6.** The droplet size and PDI values of the NEG and plain gel

Formulation code	Droplet size, nm	PDI
NEG	175.8	0.19
Plain gel	1241	0.31

Zeta potential (*ζ*) is defined as the potential difference between the surface of a tightly bound layer and an electroneutral region of the system and measures the charge on the surface of dispersed globules [2]. In nanodispersion, the zeta potential denotes the strength of the attraction between nearby charged particles [65]. A higher level of zeta potential results in greater electrostatic repulsion between the particles, minimizing aggregation/flocculation and ensuring enhanced stability of the prepared product on storage [66,67]. Zeta potential values for the NE3 and NEG were -27.34 and -28.29 mV, respectively, which can confer a suitable stability for the formulation since these values are close to the range of +30 to -30 mV. This negative charge might be assigned to the drug substance or oleic acid. In addition, the pH of the NE and NEG has an impact on the surface droplet charge. If the developed NE is acidic (pH 3 to 6), a negative charge will be observed. However, a positive charge will be observed if NE is basic in nature (pH more than 7) [68]. The NEG formulation also shows a higher zeta potential due to the presence of HA, an acidic polysaccharide with a negative charge [69]. The measured zeta potential values for NE3 and NEG are displayed in the supplementary material, Figure S7.

**Figure 3.**
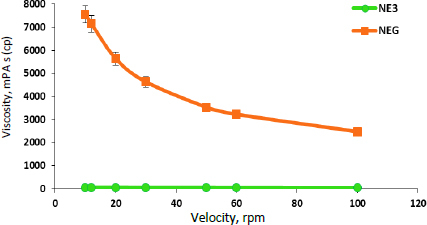
The rheogram of NE3 and NEG

The pH of the NEG was found to be 5.28 ± 0.024, which is within the physiological pH range for the skin [58]. Thus, it has less of a tendency to cause irritation of the skin. The drug content for caffeine, aminophylline, and tretinoin in NEG was 99.35 ± 0.053, 98.48± 0.302 and 98.05 ± 0.138 %, respectively. The high drug content suggests efficient drug loading and distribution throughout the gel [70]. The viscosity measurement of the NEG revealed that there was a significant (*p* ≤ 0.01) increase in the viscosity of the NEG in comparison to NE3, as shown in Figure 3. This can be attributed to the addition of hyaluronic acid, an extremely hydrophilic molecule that can absorb water, extend the solid volume by up to 1000 times its original volume, and form a viscous hydrogel [71]. The rheological behaviour is an important parameter for gel performance, which governs its spreadability, flowability, and drug release from the formulation [41,72]. Furthermore, it was observed that the NE3 rheogram displays Newtonian behaviour, where the viscosity is independent of the shear rate applied [73] while the developed NEG exhibited non-Newtonian, pseudoplastic behaviour (shear-thinning) [2]. The pseudoplastic behaviour of gel formulations is convenient for application and desirable for topical delivery due to the high apparent viscosity at low shear and hence, the low mobility of the dispersed phase, thereby keeping the gel components in a homogenous distribution [2]. Upon shear stress employment, the NEG would exhibit free flowing with decreased viscosity, allowing for easy spreading without washout or drug loss upon its application on the skin surface [74].

**Figure 4.**
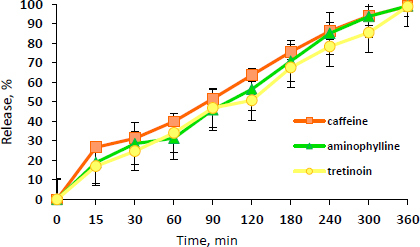
Release profiles of caffeine, aminophylline, and tretinoin from the NEG in phosphate buffer pH 7.4 with 2 % Tween 80

The release profile of caffeine, aminophylline, and tretinoin from the NEG formulation is illustrated in Figure 4.

As can be observed from Figure 4, the drugs are gradually released from the NEG, reaching approximately 100 % within 6 hours. This indicates the well-partitioning of the drugs from the gel base. Compared to the drug release from the NE3, the release of the three drugs from the NEG was significantly (*P* ≤ 0.05) lower than that of the NE3. This may be due to the higher viscosity of the NEG, which results in this mild retardation effect for 6 hours compared to 5 hours in NE3.

### *Ex vivo* permeation and skin deposition study

**Figure 5.**
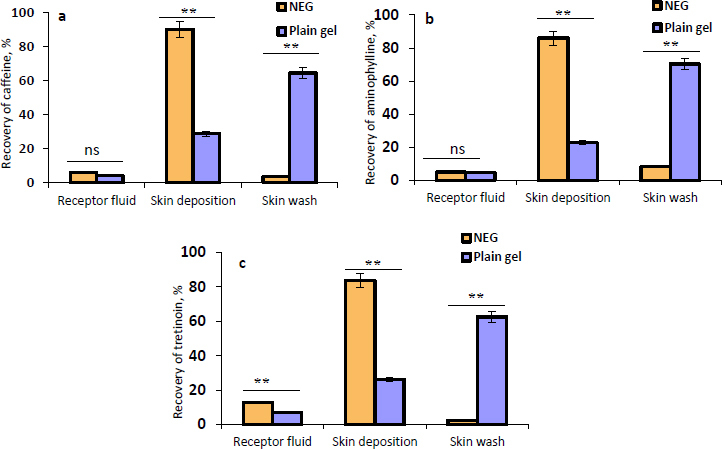
The recovery percentage of (a) caffeine, (b) aminophylline, and (c) tretinoin that appeared in the receptor fluid, deposited within the skin, or removed as a skin wash from the NEG and plain gel. Data is presented as the mean ± SD, *n* = 3, where (ns) for non-significant, and ** for *P* ≤ 0.01

The permeation of the NEG in comparison with the plain gel into/across human skin was investigated. Figures 5a, 5b, and 5c illustrate the recovery percentage of caffeine, aminophylline, and tretinoin, respectively, that permeated into the receptor fluid, deposited within the skin, or removed from the skin surface as a skin wash from the NEG, and the plain gel. The results displayed in Figure 5 apparently show that there is a significant (*P* ≤ 0.01) increase (3-fold increase) in the amount of caffeine, aminophylline, and tretinoin deposited within the skin from the NEG compared to the plain gel. Similarly, the receptor fluid from the NEG contained higher permeated amounts of the three drugs than the plain gel. However, this higher permeation into the receptor fluid was only significant with tretinoin (*P* ≤ 0.01). Additionally, it was observed that the amount of drugs that remained on the skin surface and collected by a cotton swab (skin wash) was significantly (*P* ≤ 0.01) greater in the plain gel than NEG. The larger droplet size of the plain gel may contribute to its poor skin permeation, and a higher amount, approximately 65 %, remained over the skin surface. The high permeability of NEG through the human skin compared to plain gel could be attributed to the nanodroplets of the NEG, which produced the highest surface area for drugs to permeate through the skin. In addition, the non-ionic surfactant (Tween 20) and oleic acid interact with the human skin lipids, which could increase the fluidity of skin membranes, resulting in an enhanced drug diffusion rate across skin layers [68].

### Skin pre-treatment with microneedles

Microneedles (MNs) are considered an innovative method of improving drug delivery into the skin by bypassing the *stratum corneum* barrier and creating microchannels through which the drug molecules can pass easily [75]. Thus, in this study, a microneedling pen was employed to enhance NEG delivery into the skin since this microneedling device can be easily applied to a large area of the skin, such as in the case of cellulite. One of the earliest and simplest microneedle-based approaches to promote intradermal drug delivery is via the poke-and-patch method [20,22,76].

**Figure 6.**
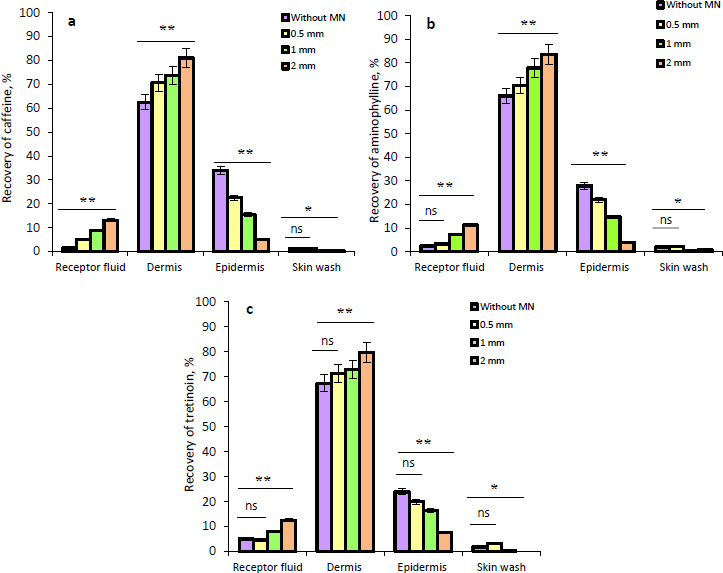
The recovery percentage of (a) caffeine, (b) aminophylline, and (c) tretinoin that determined in the receptor fluid, deposited in the dermis and epidermis, or removed as a skin wash from the NEG without and with MNs at varying lengths of 0.5, 1, and 2 mm. Data is presented as the mean ± SD, *n* = 3, where (ns) for non-significant, * for *P* ≤ 0.05, and ** for *P* ≤ 0.01

The results of the *ex vivo* skin permeation study of caffeine, aminophylline, and tretinoin from the NEG without and with MN application at different lengths are illustrated in Figures 6a, 6b and 6c. It was observed that with the application of MNs, there was an increase in the amount of caffeine, aminophylline, and tretinoin deposited in the dermis in comparison with the permeation results without MNs. This increment in the deposited amount in the dermis was highly significant (*P* ≤ 0.01) when 0.5, 1 and, 2 mm needle lengths were used. Furthermore, it can be noticed that as the needle length increased, the amount of the drugs deposited in the epidermis decreased, while the amount in the dermis and receptor fluid increased.

This can be explained by the formation of the microchannels within the epidermis and dermis that aid in the transport of drugs into the deeper dermis layer and receptor fluid. However, the percentage of drugs that reached the receptor fluid was low, which suggested a lower tendency towards systemic absorption. Since cellulite is a dermal disturbance in fatty tissue that primarily affects the dermis layer, the increase in drugs’ deposition within the dermis due to skin pre-treatment with MNs has a potential beneficial effect as a topical treatment; therefore, the treatment that maintains the drugs in the dermis will provide the most benefit for the treatment of cellulite. In addition, using the microneedling technique could assist in improving skin appearance through the stimulation of collagen and elastin production in the dermis as a result of a natural healing mechanism.

### Skin irritation test and stability study of the nanoemulgel

**Figure 7.**
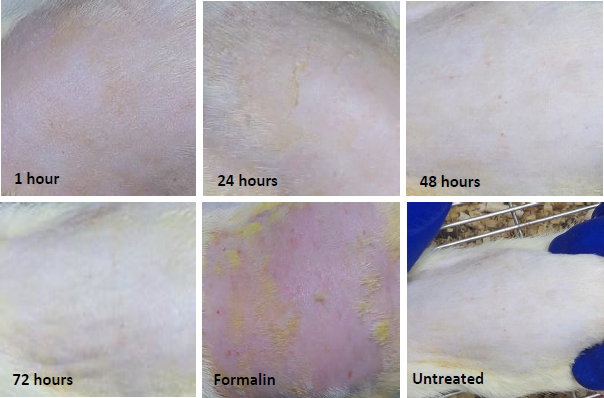
Images of the skin irritation test performed by applying of NEG on the rat’s skin at 1, 24, 48, and 72 h, in addition to the images of formalin (+ve control), and untreated skin area (-ve control)

The irritation test results showed that the NEG application sites only demonstrated mild erythema and no oedema after 1 hour and 24 hours. This erythema was completely cleared up after 48 and 72 hours, and the skin resembled the untreated area, as shown in Figure 7. In contrast, formalin caused severe erythema and oedema, indicating that NEG is a non-irritant formulation compared to formalin.

In terms of stability, the NEG demonstrated suitable short-term stability at various temperatures. The visual examination exhibited no drug precipitation, phase separation, colour change, or flocculation. In addition, the changes in droplet size, PDI, and drug content values during the storage period lie within the acceptable range. However, a longer stability study is required to confirm its stability at various storage conditions. Table 7 displays the short-term stability results.

**Table 7.** Stability study results of droplet size, PDI, pH, and drug content for the NEG during the three months storage period

*t* / °C	Time, month	Droplet size, nm	PDI	pH	Drug content, %		
					Caffeine	Aminophylline	Tretinoin
	0	175.8	0.19	5.28	99.35	98.48	98.05
4	1	151.4	0.33	5.26	99.01	98.33	97.91
	2	146.7	0.39	5.23	98.93	98.04	97.77
	3	145.3	0.38	5.22	98.84	97.97	97.71
25	1	154.2	0.41	5.29	98.79	98.26	97.36
	2	151.2	0.41	5.37	98.63	98.19	97.29
	3	142.1	0.34	5.33	98.14	98.07	97.22
40	1	154.8	0.40	5.17	97.49	97.97	96.55
	2	150.2	0.42	4.86	97.22	97.61	96.41
	3	146.1	0.41	4.66	97.00	97.39	96.21

## Conclusions

This study demonstrated unprecedentedly the development of a new cosmeceutical nanoemulgel containing triple therapy as a topical anticellulite product. It showed higher permeation and skin deposition than the plain gel due to its nanosized droplets and permeation enhancement components, including the oil and surfactants. Furthermore, the application of microneedles as a skin pre-treatment is an effective technique for improving the permeation of drugs through the skin and increasing drug deposition within the dermis. Simultaneously, they may contribute to the enhancement of the skin's appearance by stimulating the synthesis of collagen and elastin in the dermis as a natural healing process. As a result, the current study may provide an innovative approach for efficient topical cellulite treatment. Nevertheless, additional *in vivo* tests are needed to validate the clinical safety and efficacy of this treatment approach.

Supplementary material Additional data are available at https://pub.iapchem.org/ojs/index.php/admet/article/view/2461, or from the corresponding author on request.
