# Supplementary material for: Combined approach of nanoemulgel and microneedle pre-treatment as a topical anticellulite therapy
Source: ADMET DMPK. 2024 Nov 10;12(6):903–23. doi: 10.5599/admet.2461 (PMC11661800; doi:10.5599/admet.2461)
Supplement: Supplementary file 1 — Supplementary material [file ADMET-12-2461-S1.pdf]

ADMET & DMPK 12(6) (2024) S13-S15

Open Access : ISSN : 1848-7718

http://www.pub.iapchem.org/ojs/index.php/admet/index

Supplementary material to

Combined approach of nanoemulgel and microneedle pre-treatment as a topical anticellulite therapy

Hiba Imad Hameed and Mohammed Hussain Al-Mayahy*

Department of Pharmaceutics, College of Pharmacy, Mustansiriyah University, Baghdad, Iraq

ADMET & DMPK **12(6)** (2024) -000; https://doi.org/10.5599/admet.2461

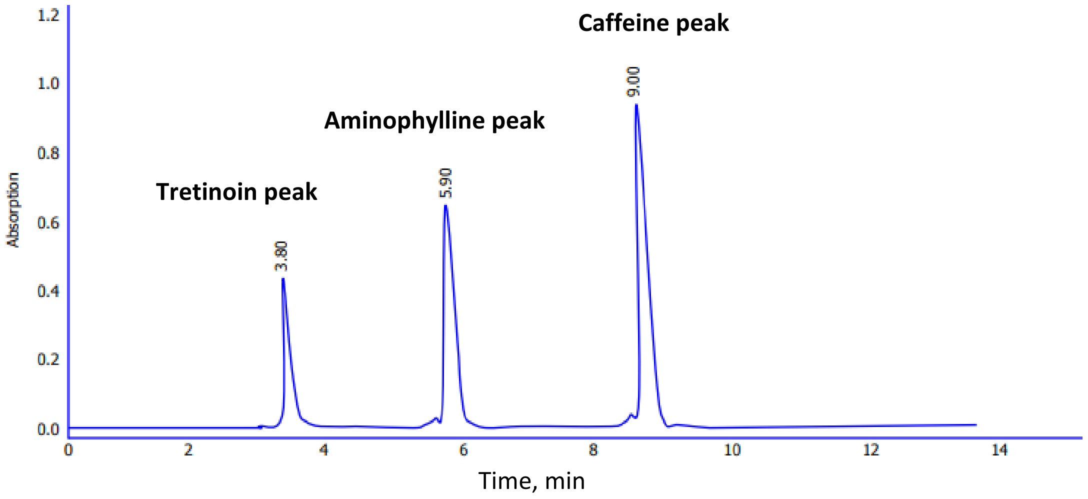

Figure S1. The HPLC chromatogram of the mixture of three drugs (caffeine, aminophylline, and tretinoin), showing the retention time of each drug

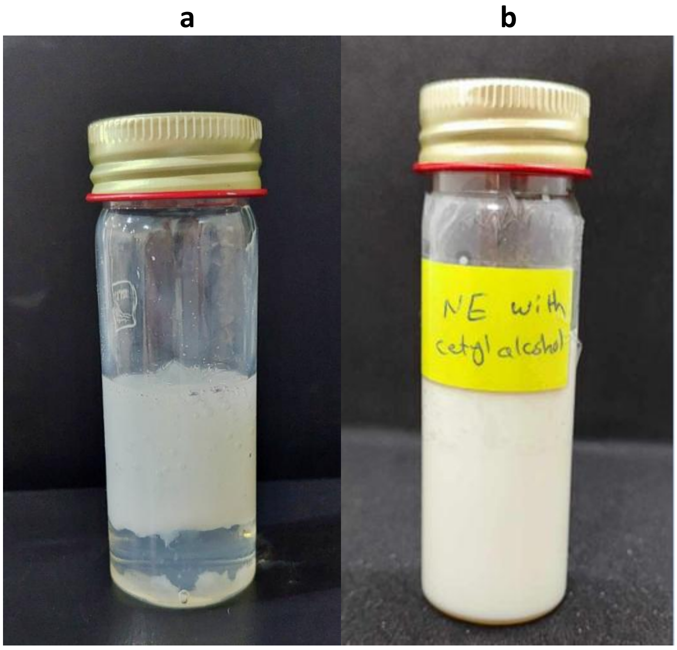

Figure S2. Images of the prepared NE formulations, a - without cetyl alcohol, and b - with cetyl alcohol.

a **b**

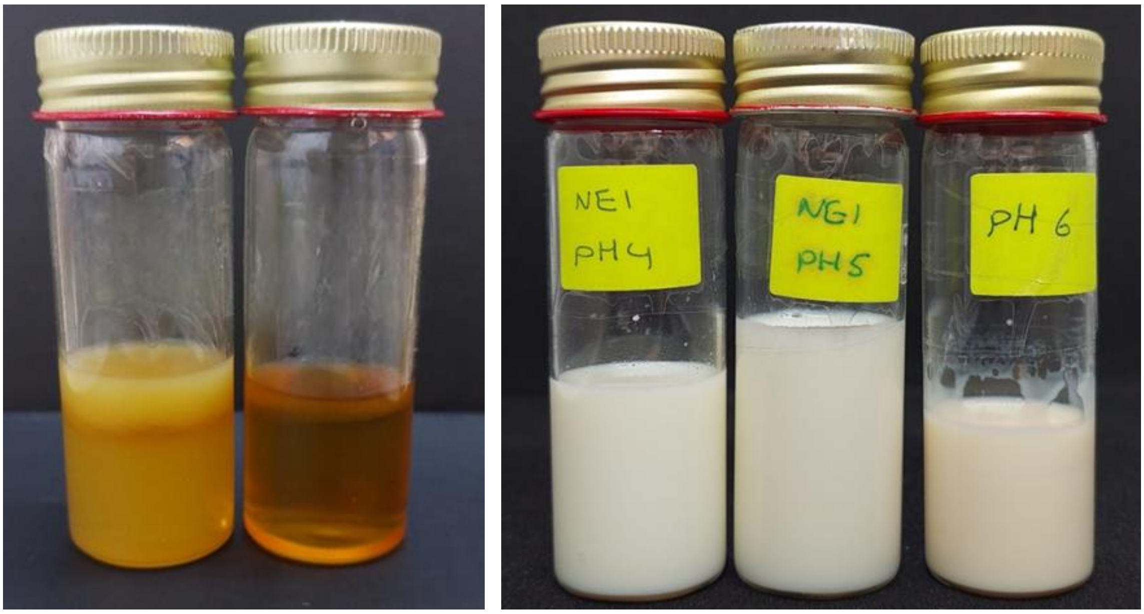

Figure S3. Images of the NE formulations prepared at a - pH 8, and b - pH 4, pH 5 and pH 6.

a **b** **c**

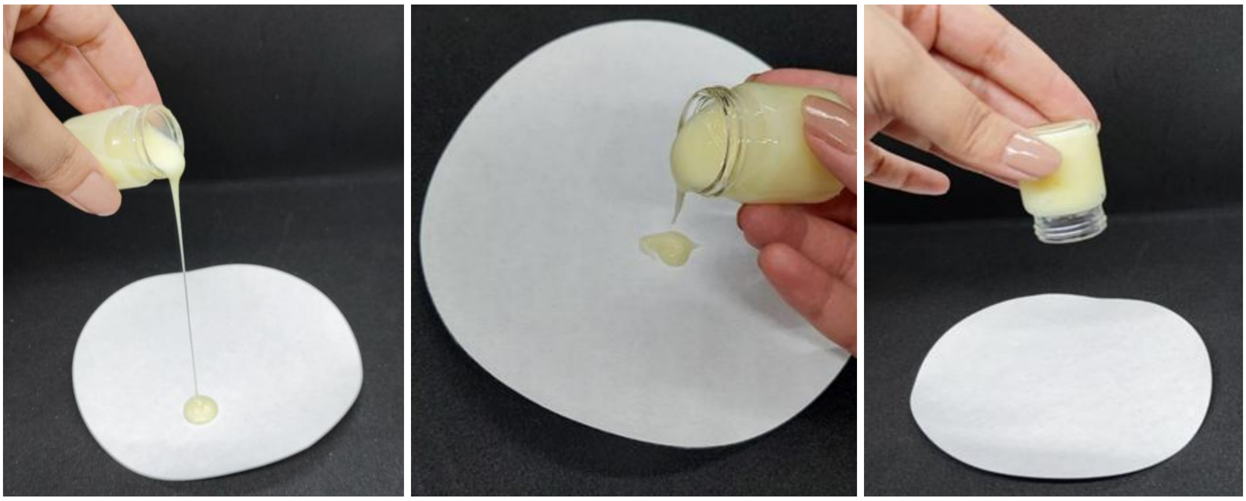

Figure S4. Images of the nanoemulgels prepared at different concentrations of HA (a) 1; (b) 1.5 and (c) 2 wt.%

a **b** **c**

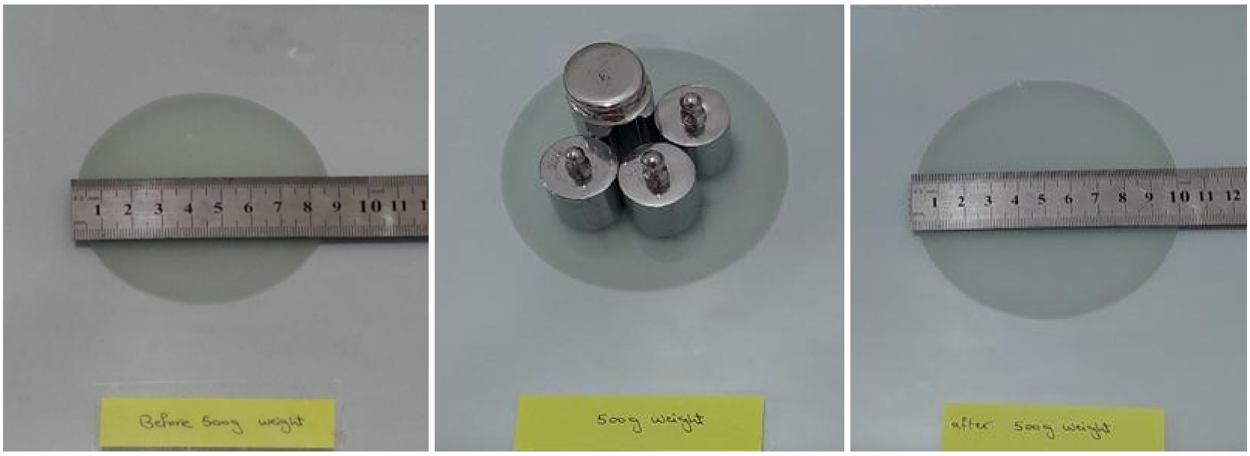

Figure S5. Images of the spreadability test of the nanoemulgel a - before the application of 500 g weight, b - during the application of 500 g weight, and c - after 5 minutes of the application of 500 g weight

(cc) BY

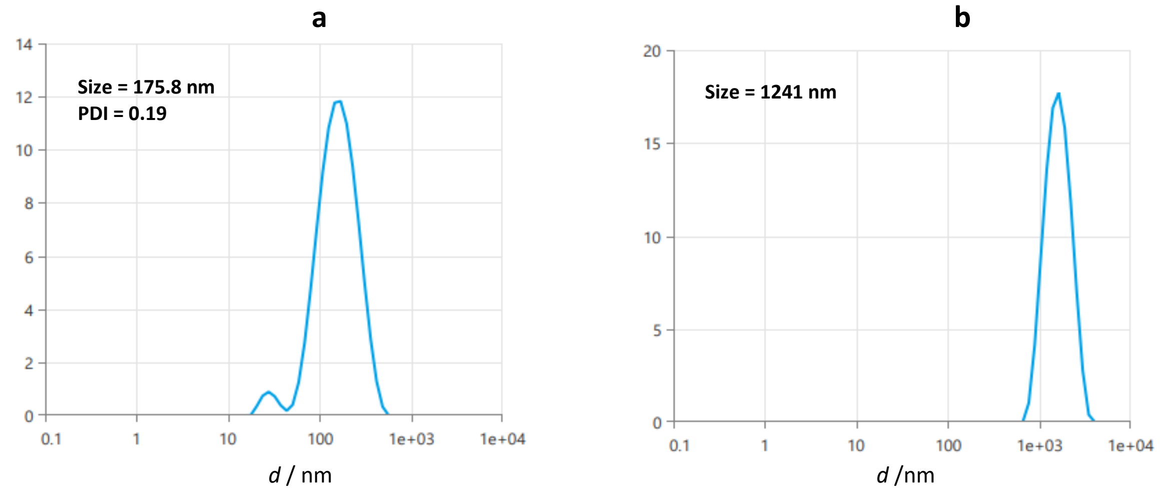

Figure S6. The droplet size distribution of a - NEG, and b - plain gel

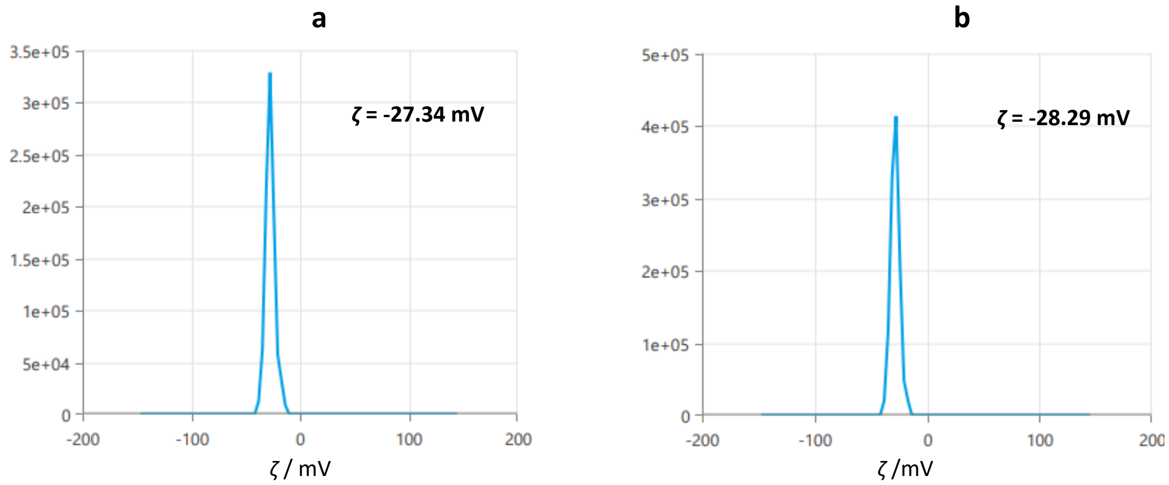

Figure S7. The measured zeta potential values for a - NE3, and b - NEG.
