# Supplementary material for: Combined approach of nanoemulgel and microneedle pre-treatment as a topical anticellulite therapy
Source: ADMET DMPK. 2024 Nov 10;12(6):903–23. doi: 10.5599/admet.2461 (PMC11661800; doi:10.5599/admet.2461)
Supplement: Supplementary file 2 — Supplementary material [file ADMET-12-2461-S1.docx]

*ADMET & DMPK 12(6) (2024) S13-S15*

*
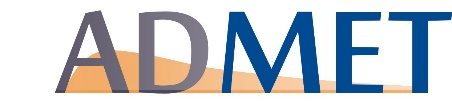
***Open Access : ISSN : 1848-7718**[***http://www.pub.iapchem.org/ojs/index.php/admet/index***](http://www.pub.iapchem.org/ojs/index.php/admet/index)

Supplementary material to

**Combined approach of nanoemulgel and microneedle pre-treatment as a topical anticellulite therapy**

Hiba Imad Hameed and Mohammed Hussain Al-Mayahy*

*Department of Pharmaceutics, College of Pharmacy, Mustansiriyah University, Baghdad, Iraq*

ADMET & DMPK **12(6)** (2024) -000; <https://doi.org/10.5599/admet.2461>


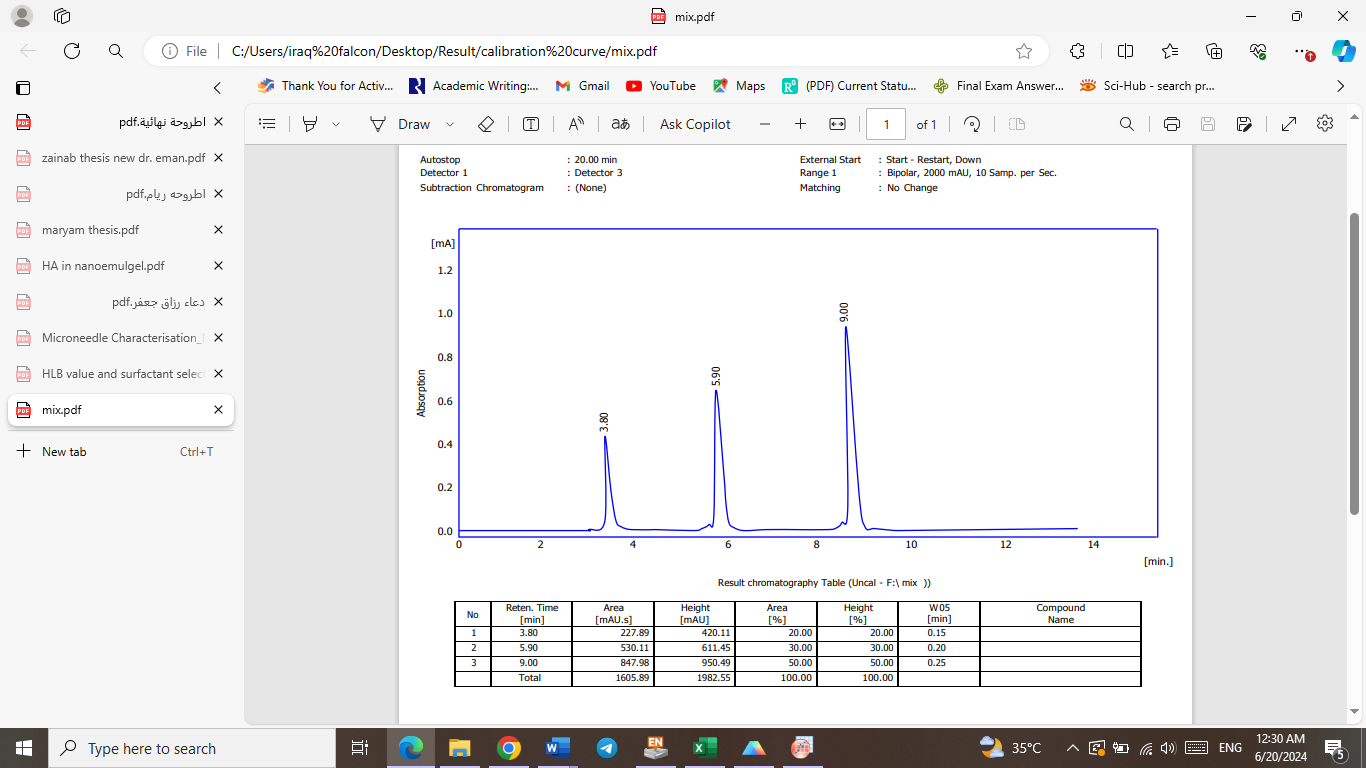


**Caffeine peak**

**Aminophylline peak**

**Tretinoin peak**

Time, min

**Figure S1.** The HPLC chromatogram of the mixture of three drugs (caffeine, aminophylline, and tretinoin), showing the retention time of each drug

**a b**


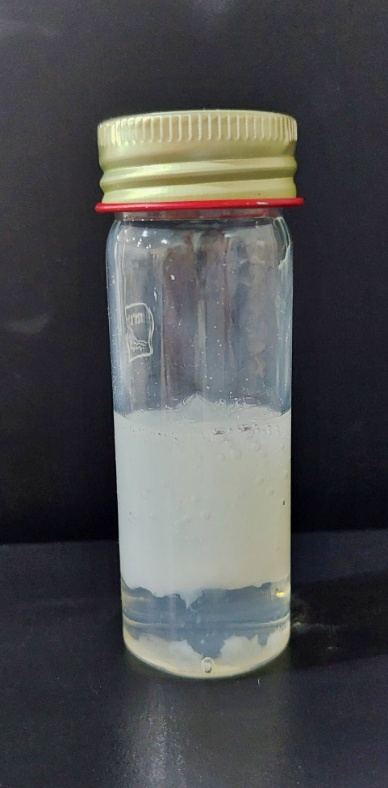
**
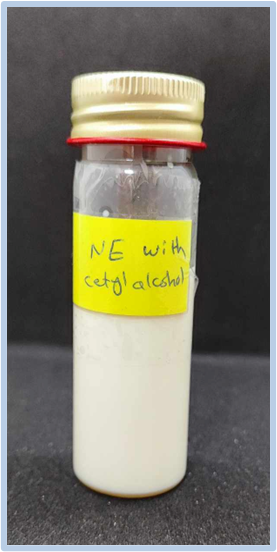
**

**Figure S2.** Images of the prepared NE formulations, a - without cetyl alcohol, and b - with cetyl alcohol.

**a b**

**
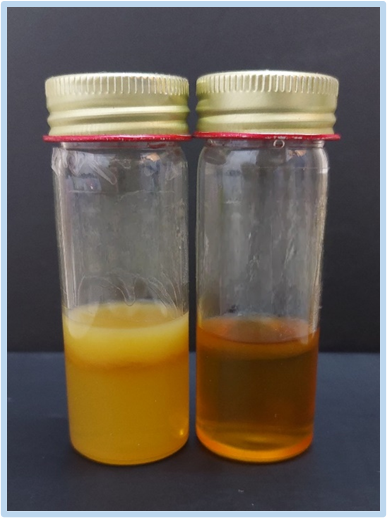

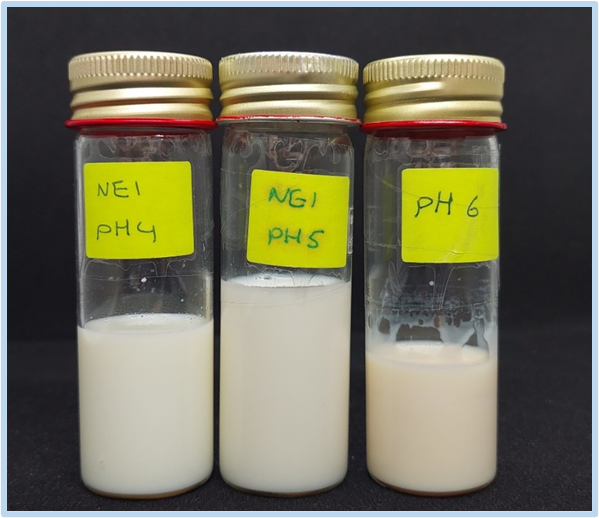
**

**Figure S3.** Images of the NE formulations prepared at a - pH 8, and b - pH 4, pH 5 and pH 6.

**a b c**


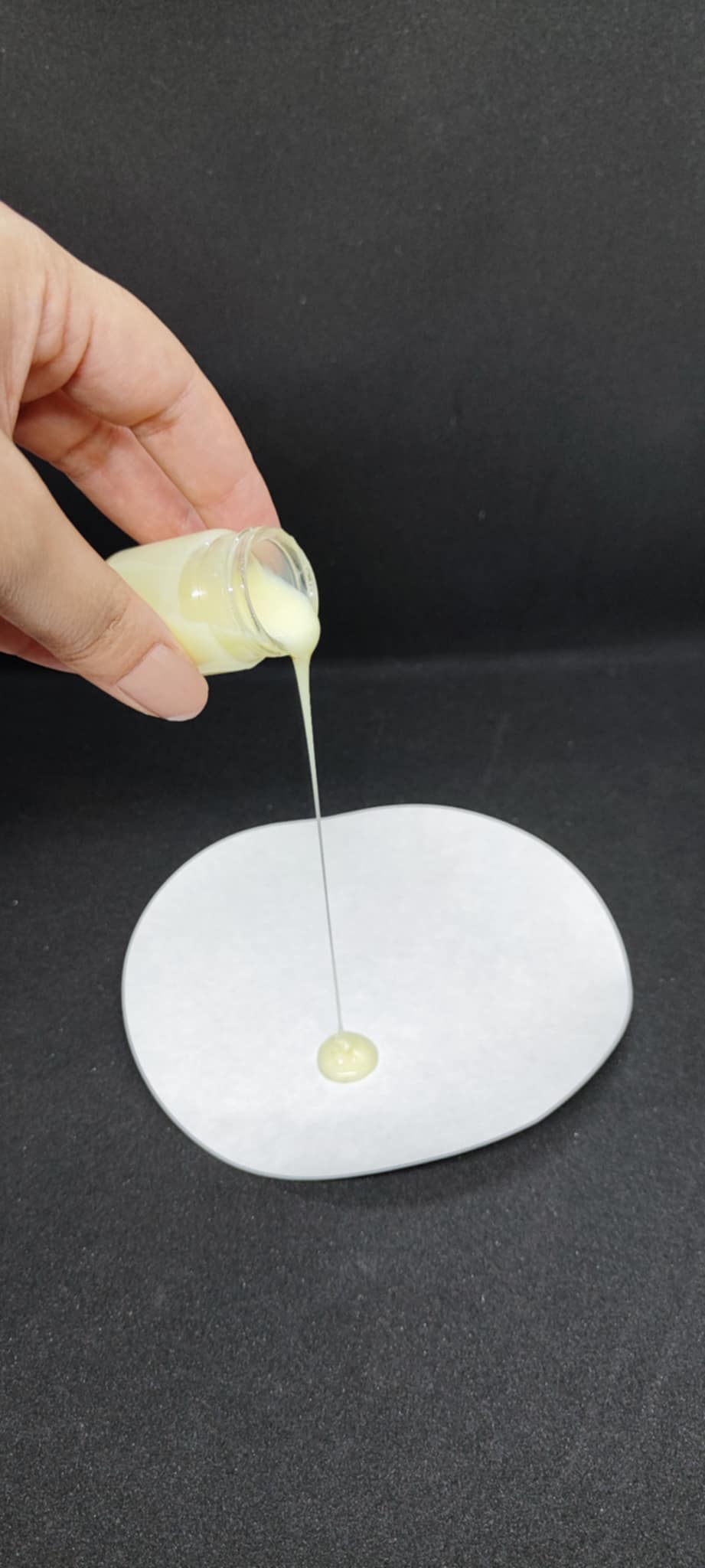

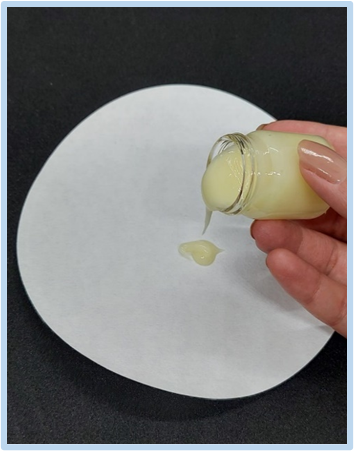

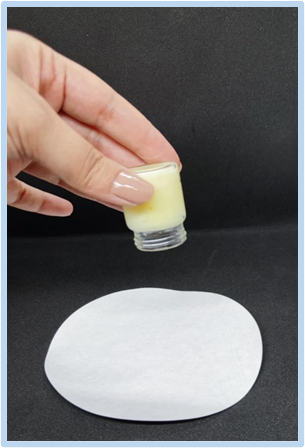


**Figure S4.** Images of the nanoemulgels prepared at different concentrations of HA (a) 1; (b) 1.5 and (c) 2 wt.%

**a b c**


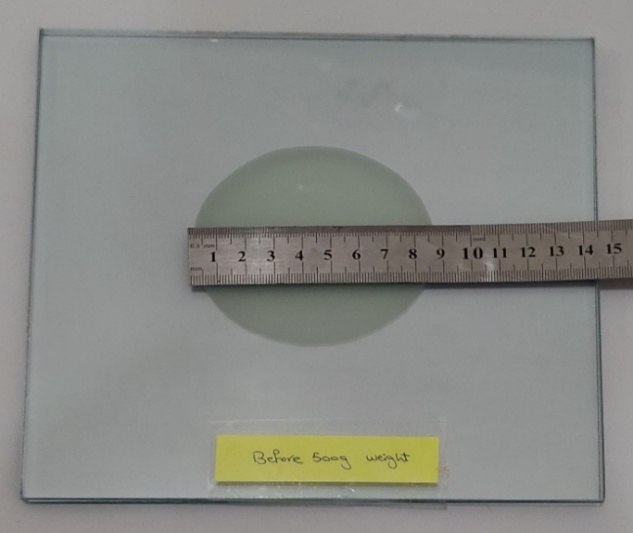

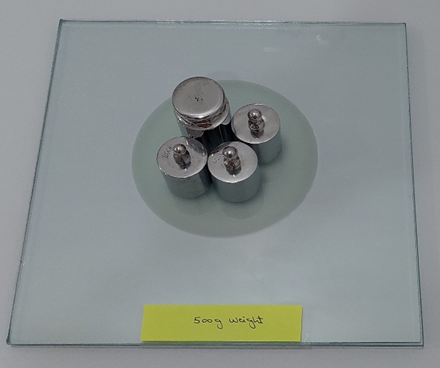

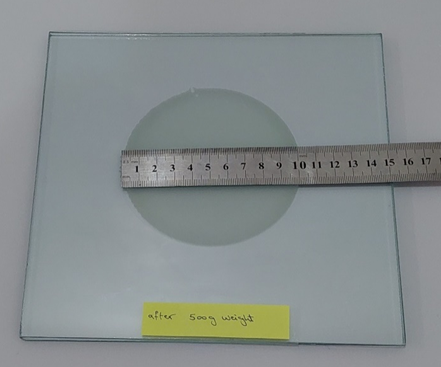


**Figure S5.** Images of the spreadability test of the nanoemulgel a - before the application of 500 g weight,
b - during the application of 500 g weight, and c - after 5 minutes of the application of 500 g weight

**a b**


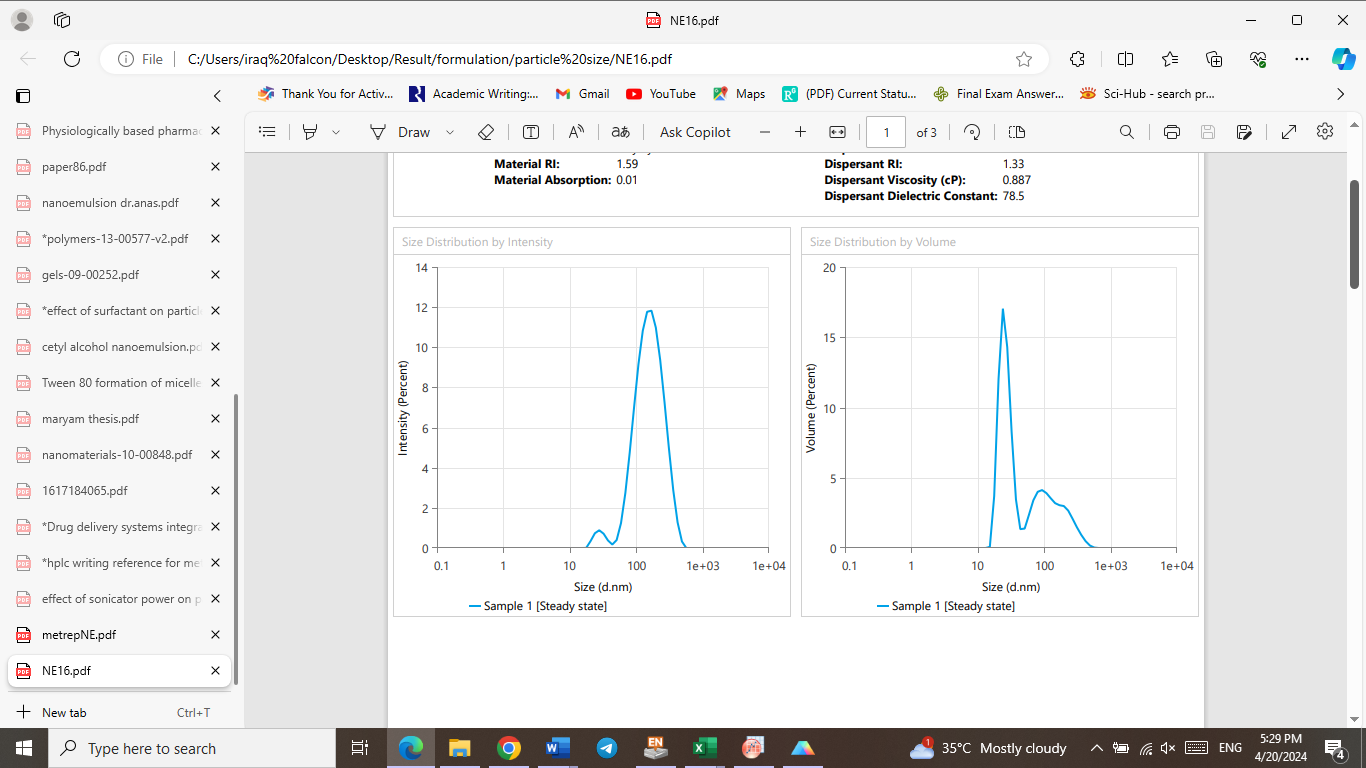


**Size = 175.8 nm**

**PDI = 0.19**


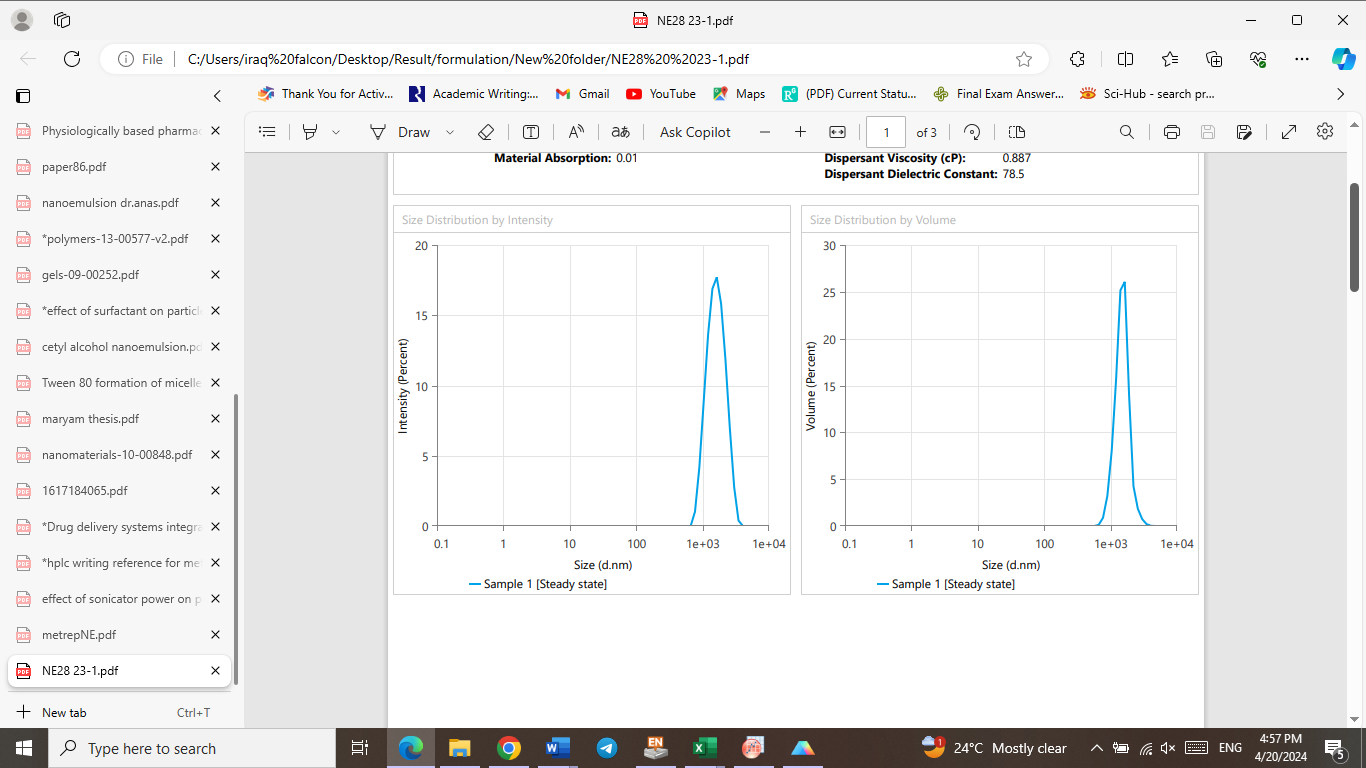


**Size = 1241 nm**

**PDI = 0.31**

*d* / nm *d* /nm

**Figure S6.** The droplet size distribution of a - NEG, and b - plain gel

**a b**


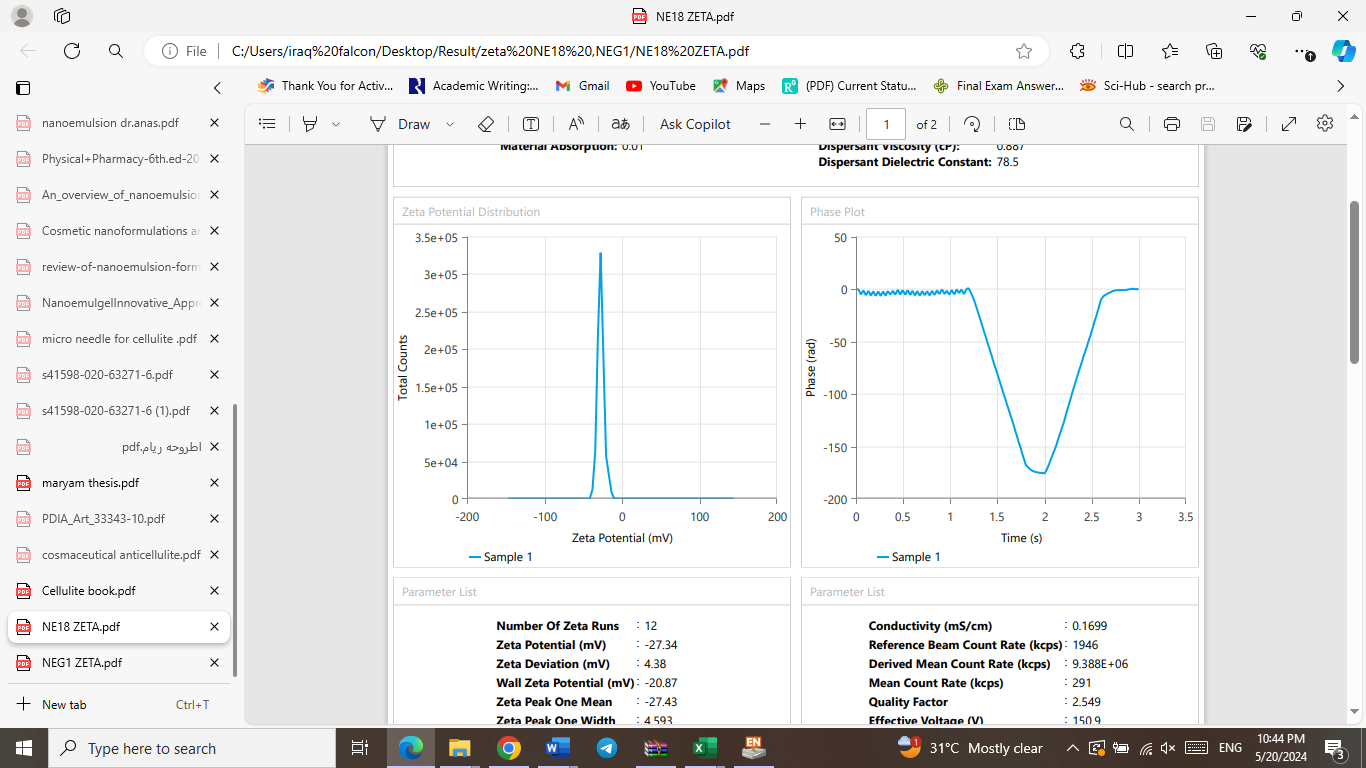


***ζ* = -27.34 mV**


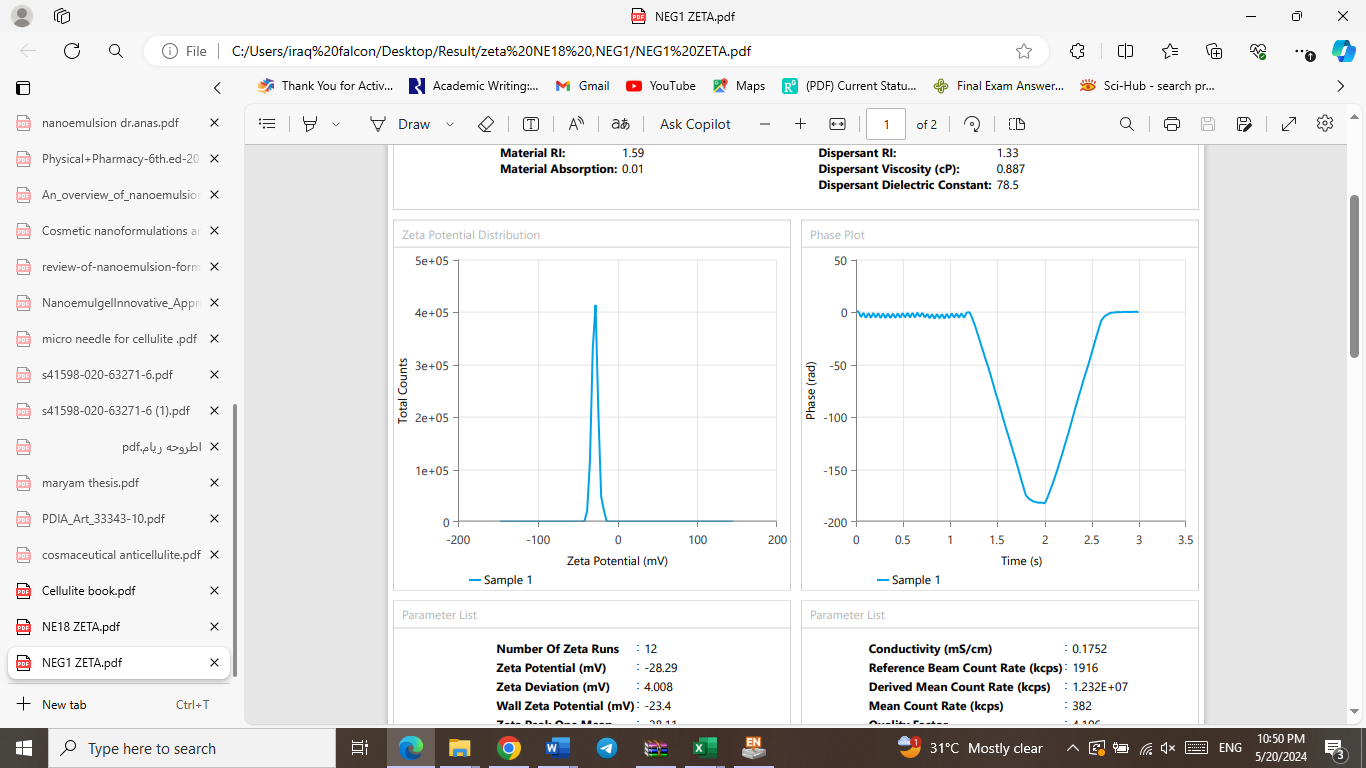


***ζ* = -28.29 mV**

*ζ* / mV *ζ* /mV

**Figure S7.** The measured zeta potential values for a - NE3, and b - NEG.
